# A predictive nomogram for lymph node metastasis in part-solid invasive lung adenocarcinoma: A complement to the IASLC novel grading system

**DOI:** 10.3389/fonc.2022.916889

**Published:** 2022-08-15

**Authors:** Zhaoming Gao, Xiaofei Wang, Tao Zuo, Mengzhe Zhang, Zhenfa Zhang

**Affiliations:** ^1^ Department of Lung Cancer Surgery, Tianjin Medical University Cancer Institute and Hospital, National Clinical Research Center for Cancer, Key Laboratory of Cancer Prevention and Therapy, Tianjin's Clinical Research Center for Cancer, Tianjin, China; ^2^ Department of Thoracic Surgery, Binzhou People’s Hospital Affiliated to Shandong First Medical University, Binzhou, China; ^3^ Department of Thoracic Surgery, The Central Hospital of Wuhan, Tongji Medical College, Huazhong University of Science and Technology, Wuhan City, China

**Keywords:** IASLC, part-solid, lung adenocarcinoma, metastasis, nomogram

## Abstract

**Background:**

The International Association for the Study of Lung Cancer (IASLC) proposed a novel grading system for invasive lung adenocarcinoma, but lymphatic invasion was not evaluated. Meanwhile, the scope of lymph node dissection in part-solid invasive lung adenocarcinoma (PSILA) is still controversial. Therefore, this study aims to explore preoperative risk factors for lymph node metastasis in PSILA, to provide reference for intraoperative dissection of lymph nodes.

**Methods:**

From 2018 to 2020, clinical data of patients (stage cN0) consecutively diagnosed as PSILA were retrospectively analyzed and classified according to the novel grading system. Logistic regression was conducted to screen the clinicopathological factors of lymph node metastasis in PSILA.

**Results:**

A large cohort of 960 patients with PSILA who underwent lobectomy or sub-lobectomy were enrolled. By logistic regression analyses, solid part size, bronchial cutoff sign, spiculation, and carbohydrate antigen 199 (CA199) were eventually identified as independent risk factors for lymph node metastasis, based on which a nomogram was built to preoperatively predict the risk of lymph node metastasis [area under the receiver operating characteristic curve (AUC)=0.858; concordance index = 0.857; best cutoff, 0.027]. This suggests that intraoperative systematic lymph node dissection is recommended when the predicted risk value exceeds 0.027. Reproducibility of the novel grading system was verified.

**Conclusions:**

The novel IASLC grading system was applicative in real world. The nomogram for preoperative prediction of lymph node metastasis may provide reference for the lymph node dissection strategy during PSILA surgeries.

## Introduction

According to the latest global cancer statistics, lung cancer ranks first in cancer-related deaths worldwide, with an 18% mortality rate. This is mainly due to the fact that most patients are already at a non-early stage when diagnosed, and the lung cancer itself has a high rate of lymph node metastasis (1). Fortunately, with the improvement of human health awareness and the promotion of thin-slice computed tomography (CT), more and more early-stage lung cancers have been detected, which usually appears in the form of pure ground glass opacity (GGO, no solid component) or part-solid nodules (ground glass and solid component) of lung adenocarcinoma.

At present, it is generally believed that part-solid invasive lung adenocarcinoma (PSILA), a special subtype of early lung adenocarcinoma, is between the indolent pure GGO and the more aggressive pure solid nodules (2). Therefore, scholars around the world have carried out different explorations on the methods of lymph node dissection during PSILA surgeries. Li et al. found that no lymph node metastasis occurred in GGO-predominant PSILA, and lymph node metastasis in solid-predominant PSILA was also rare, even if the tumor was >3 cm. Therefore, it might not be necessary to perform mediastinal lymph node dissection for PSILA (3). Hattori et al. compared the prognostic differences in 462 patients with stage I PSILA who underwent systematic lymph node dissection, lobe-specific lymph node dissection, and hilar lymph node dissection only. They found that the method of lymph node dissection was not associated with the prognosis of patients (4). Another multicenter study of 565 patients with early-stage lung cancer also yielded similar results after Propensity Score Matching (5). However, another retrospective cohort study of 825 patients found that compared with the 0% lymph node metastasis rate of pure GGO, the metastasis rate of 6.9% in PSILA was not negligible. Therefore, systematic lymph node dissection is recommended (6). Obviously, more powerful research evidence is urgently needed for the choice of intraoperative lymph node dissection in PSILA.

Lung adenocarcinomas are histologically heterogeneous and present with multiple combinations of patterns and proportions. Rare subtypes, such as cribriform and fused glands, are often characterized by increased levels of atypia, enhanced mitosis, and vascular infiltration (7, 8), which have been shown to have the same poor prognosis as solid and micropapillary predominant adenocarcinoma (9, 10). Therefore, the International Association for the Study of Lung Cancer (IASLC) issued the novel grading system for invasive lung adenocarcinoma [based on predominant histologic plus high-grade patterns (20%)], removing complex glandular patterns (cribriform and fusion glands) from the previous acinar pattern and combining them with micropapillary, solid collectively referred to as high-grade patterns. Compared to the conventional predominant pattern-based groups, the IASLC novel grading system could more practically predict recurrence-free survival and overall survival in invasive adenocarcinoma (11, 12). However, it must be noted that the influence of lymphatic invasion on patient survival was not evaluated in this new system.

**Figure 1:**
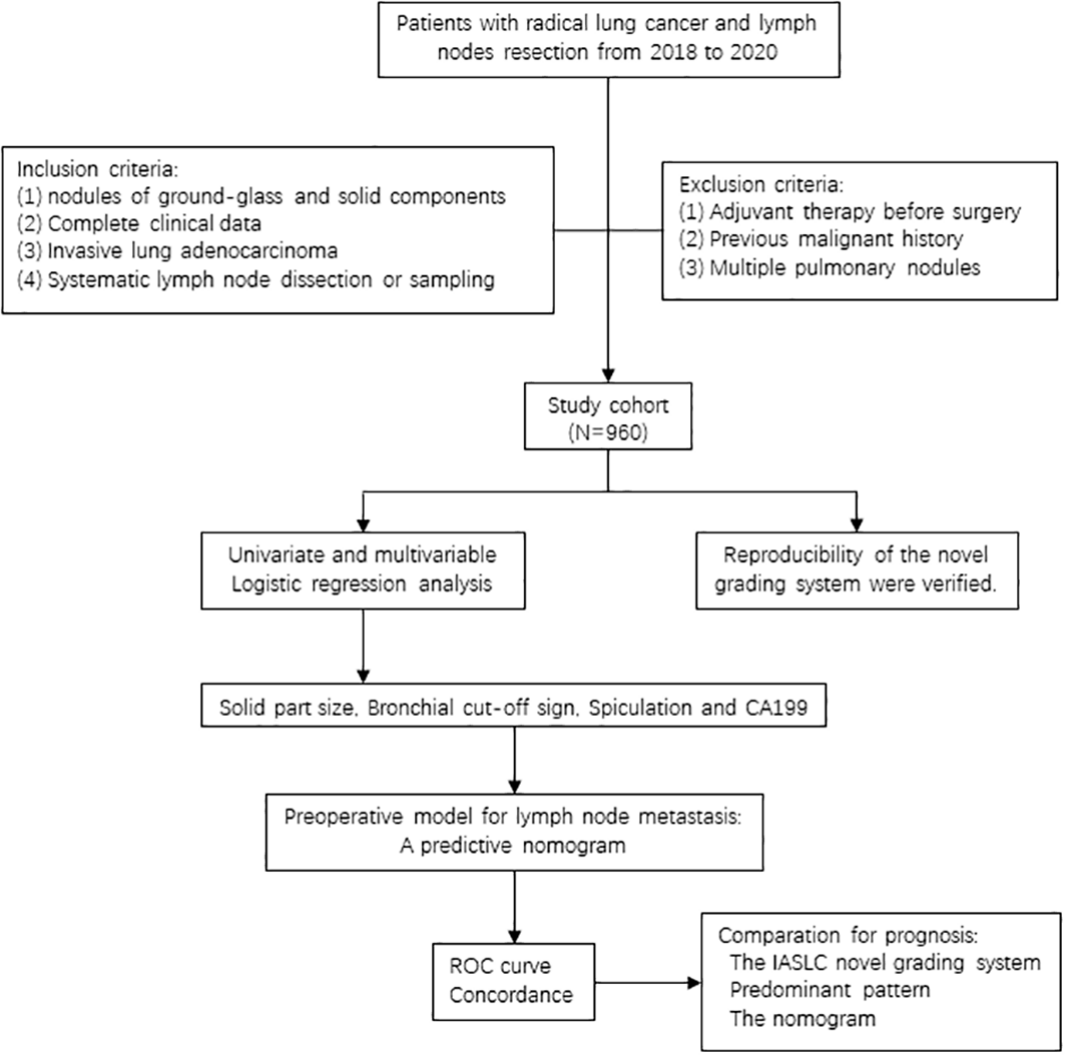
Flow diagram of this study.

As we all know, lymphatic tract is the main way of lung cancer metastasis, and its threat to the survival of patients cannot be ignored. Therefore, considering the current global disagreement on PSILA lymph node dissection and the gap of lymphatic invasion evaluation in the IASLC novel grading system, this study aims to use the real-world data to validate the reproducibility of the novel grading system and explore preoperative risk factors for PSILA lymph node metastases, hoping to provide a reference for PSILA intraoperative lymph node dissection. The flow diagram of this study is shown in **Figure 1**.

## Materials and methods

### Clinical data

We collected the consecutive data of patients (stage N0) with PSILA who underwent radical resection and lymphadenectomy in Tianjin Medical University Cancer Institute & Hospital from January 2018 to December 2020. Clinicopathological data enrolled included the following (1): baseline information, namely, age, gender, smoking history, and operative method (2); CT features, namely, tumor location, tumor size, solid part size, and consolidation to tumor ratio (CTR) and signs like bronchial cutoff, spiculation, air bronchogram, cavitary, vocule, lobulation, pleural retraction, and vascular cluster (3); pathological data, namely, histological subtype, lymph node metastasis status, vascular tumor thrombus, and spread through air spaces (STAS) (4); tumor marker, namely, carcinoembryonic antigen (CEA), carbohydrate antigen 199 (CA199), and tissue polypeptide specific antigen (TPS); and (5) drive gene alternation of EGFR, ALK, and KRAS.

The inclusion criteria were as follows (1): lung nodules of patients contain both ground-glass and solid components (2), complete clinical data are available (3), histopathology belongs to invasive lung adenocarcinoma, and (4) systematic lymph node dissection or sampling. Exclusion criteria were the following (1): received neoadjuvant therapy before surgery (2), previous malignant history, and (3) multiple pulmonary nodules. Finally, 960 patients were enrolled in this study.

Tumor–node–metastasis (TNM) staging according to the eighth edition of the American Joint Committee on Cancer staging manual and clinical T staging are based on solid component size (13). The histological subtypes of lung adenocarcinoma were classified according to the 2015 WHO classification (11). Pathology grades of invasive pulmonary adenocarcinoma were defined in line with the 2020 IASLC novel grading system (12). All pathology reports and imaging features were identified by one junior physician and one intermediate physician and confirmed after review by two senior physicians.

Nomogram is a scoring standard based on the regression coefficients of all variables in multivariate analysis. For the value of each indicator in the figure, a score can be obtained by comparing the scale above the figure. Finally, the scores of all indicators are summed and converted into the probability of each patient’s outcome event through the function (14). In this study, we aimed to build a nomogram based on diverse preoperative factors, providing a reference for intraoperative lymph node dissection.

### Statistical analysis

All statistical analysis and graphic were performed using SPSS 21.0 and R 3.63 software. Categorical data were presented as frequencies with proportions and were analyzed using χ^2^ or Fisher’s exact test. Continuous data were expressed as means ± standard deviation and were compared between groups using the Student’s t-test or the Mann–Whitney U test. Logistic regression analyses were used to screen risk factors associated with lymph node metastasis and used for modeling. Area under the receiver operating characteristic curve (AUC) and concordance index (C-index) were used to evaluate the predictive ability of related factors for lymph node metastasis. The best predictive cutoff was determined by the Youden Index. Two-tailed *p*-values <0.05 were defined as statistically significant.

## Results

### Clinicopathological factors of lymph node metastasis

As shown in **Table 1**, no significant association was found between age, gender, smoking history, tumor location, operative method, drive gene mutations (ALK/EGFR/KRAS), and lymph nodes (all *p* > 0.05). The overall metastatic rate of the 960 patients with PSILA was 3.02% (29/960), which was consistent with the results of current studies. In addition, compared with the previous classification based on predominant patterns, patients of grade 3 based on the IASLC novel system had “increased,” from 20/960 (2.1%) to 171/960 (17.8%), which was due to the novel system; the high-grade pattern includes patients with complex glands previously classified as acinar subtype. Obviously, this is bound to bring more favorable attention and treatment to these “lucky” patients due to the poor prognosis of complex glandular patterns. Meanwhile, there was no lymph node metastasis in stage IA–B patients, and patients with lymph node metastasis were mainly in stage IIB to IIIA (IIB, 8/9; IIIA, 21/21); the difference was statistically significant (*p* < 0.001). As expected, tumor size, solid part size, and CTR were significantly larger in patients suffering lymph node metastasis than those without lymph node metastasis (all *p* < 0.001).

**Table 1:** Relationship between clinicopathologic characteristics and lymph node metastasis in all patients (N = 960).

Lymph node metastasis			
Variables	Negative	Positive	*p*-value
**Gender**			0.848
Male	342	10	
Female	589	19	
**Age, year**			0.610
	58.86 ± 8.322	59.66 ± 6.847	
**Smoking history**			0.543
Never	284	7	
Ever	647	22	
**Operative procedure**			0.144
VATS	809	24	
R-VATS	105	3	
Open	12	1	
Other	5	1	
**High-grade patterns, %**			<0.001
	7.959 ± 11.429	29.483 ± 21.931	
**IASLC grading**			<0.001
Grade 1	462	3	
Grade 2	321	3	
Grade 3	148	23	
**Predominant pattern**			<0.001
Low	494	6	
Medium	420	20	
High	17	3	
**Tumor stage**			<0.001
IA	901	0	
IB	23	0	
IIA	6	0	
IIB	1	8	
IIIA	0	21	
**Tumor size**			<0.001
	21.344 ± 8.960	31.586 ± 11.963	
**Solid part size**			<0.001
	12.598 ± 7.566	24.483 ± 10.384	
**CTR, %**			<0.001
	58.945 ± 21.967	76.905 ± 13.862	
**Tumor location**			0.907
Right upper lobe	364	11	
Right middle lobe	47	1	
Right inferior lobe	147	3	
Left upper lobe	251	10	
Left inferior lobe	122	4	
**Bronchial cutoff sign**			0.002
Present	16	4	
Absent	915	25	
**Spiculation**			0.039
Present	491	21	
Absent	440	8	
**Air-bronchogram sign**			0.696
Present	336	9	
Absent	595	20	
**Cavitary sign**			1.000
Present	22	0	
Absent	909	29	
**Vocule sign**			0.197
Present	278	6	
Absent	653	23	
**Lobulation sign**			0.241
Present	742	26	
Absent	189	3	
**Pleural retraction sign**			0.441
Present	562	20	
Absent	369	9	
**Vascular cluster sign**			0.089
Present	409	8	
Absent	522	21	
**STAS**			1.000
Present	57	2	
Absent	874	27	
**Vascular tumor thrombus**			0.001
Present	0	2	
Absent	931	27	
**ALK mutation**			1.000
Present	10	0	
Absent	499	13	
**EGFR mutation**			0.077
Present	152	11	
Absent	63	0	
**Kras mutation**			0.487
Present	12	1	
Absent	203	10	
**TPS**			0.974
	59.382 ± 58.322	60.134 ± 48.838	
**CA199**			0.021
	12.382 ± 8.542	16.256 ± 9.732	
**CEA**			0.172
	4.014 ± 33.490	12.874 ± 21.077	

R-VATS, robotic video-assisted thoracoscopic surgery; IASLC, International Association for the Study of Lung Cancer; CTR, consolidation to tumor ratio; STAS, spread through air spaces; TPS, tissue polypeptide specific antigen; CA199, Carbohydrate antigen199, a non-specific mucin type carbohydrate protein tumor marker; CEA, carcinoembryonic antigen.

**Figure 2:**
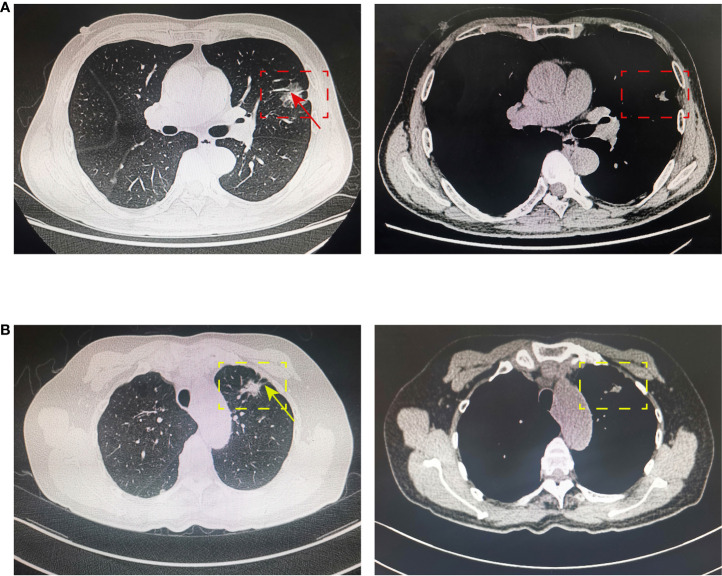
Bronchial cutoff and spiculation sign on CT. **(A)** The bronchial cutoff sign of PSILA is shown in the pulmonary window (left) and mediastinal window (right). The red box shows the location of the tumor, and the red arrow shows the classical bronchial cutoff sign. **(B)** The spiculation sign of PSILA is shown in the pulmonary window (left) and mediastinal window (right). The yellow box shows the location of the tumor, and the yellow arrow shows the classical spiculation sign. .

For CT features, patients with positive lymph node metastasis are more likely to have bronchial cutoff (*p* = 0.002) and spiculation sign (*p* = 0.039) on CT (**Figure 2**), and lung cancer with these two imaging features is generally considered to be more aggressive (15, 16). However, no significant differences were found between patients in the characteristic sign of air bronchogram, cavitary, vocule, lobulation, pleural retraction, and vascular cluster (all *p* > 0.05). Similarly, no significant association was found between the presence of spread through airspaces (59/960) and lymph node metastasis (*p* > 0.05).

For hematological test, CA199 in patients with lymph node metastasis (16.256 ± 9.732 U/ml) was significantly higher than that in patients without lymph node metastasis (12.382 ± 8.542 U/ml), while tissue polypeptide specific antigen (TPS) and carcinoembryonic antigen (CEA) showed no statistical difference between patients of two groups (*p* > 0.05).

### Construction and validation of a nomogram to predict lymph node metastasis

Considering the current controversy in comparing the prognostic ability of tumor size, solid part size, and CTR, multiple ROC curves of the three indicators in predicting lymph node metastasis were constructed in this study. As shown in the **Supplementary Figure S1**, tumor size (AUC=0.755, 0.668–0.843), solid part size (AUC=0.834, 0.706–0.904), and CTR (AUC=0.738, 0.668–0.808) all had a good predictive ability (all *p* < 0.001). As shown in **Table 2**, in order to construct a model for preoperative prediction of lymph node metastasis based on clinicopathological indicators, we finally selected solid part size (with the largest AUC value), bronchial cutoff sign and spiculation sign on CT, and the hematological indicator CA199, which were included in multivariate logistic regression analysis for subsequent modeling.

**Table 2:** Univariate and multivariate logistic regression analyses of risk factors for lymph node metastasis.

Predictor	Univariate analysis		Multivariate analysis	
	*p*	OR (95% CI)	*p*	OR (95% CI)
CA199	0.023	1.039 (1.005, 1.074)	0.05	1.037 (1.000, 1.076)
Spiculation	0.042	2.352 (1.031, 5.365)	0.233	1.742 (0.700, 4.334)
Solid part size	<0.001	1.123 (1.085, 1.061)	<0.001	1.113 (1.073, 1.154)
Bronchial cutoff sign	<0.001	9.150 (2.853, 29.348)	0.32	2.112 (0.484, 9.221)

**Figure 3:**
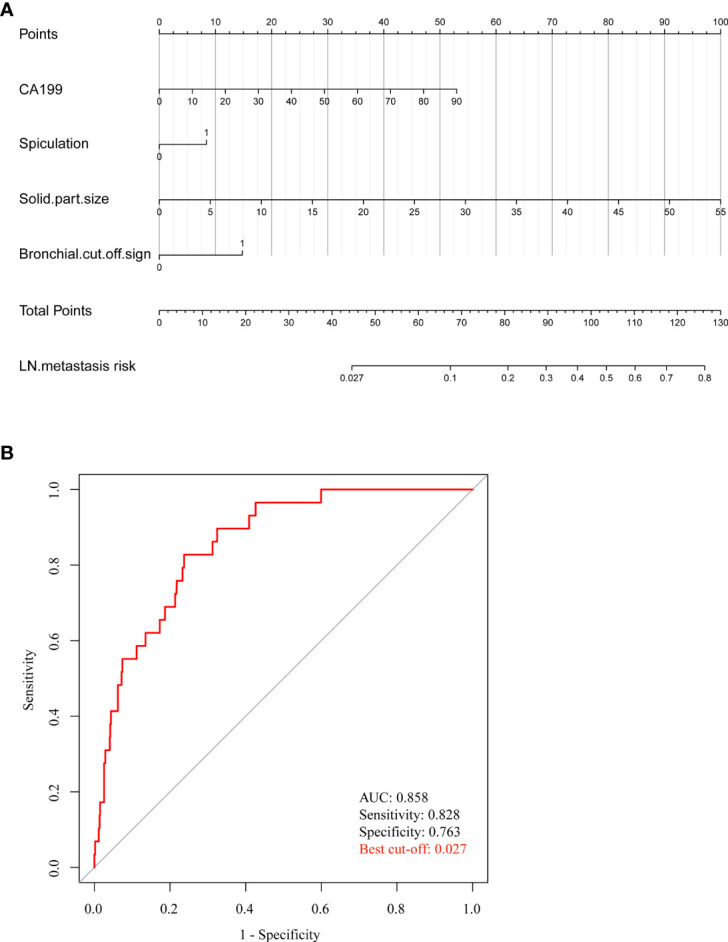
Nomogram and its ROC curve for preoperatively predicting the risk of lymph node metastasis in PSILA. **(A)** The nomogram built for preoperatively predicting the risk of lymph node metastasis. Points of four indicators (CA199, solid part size, spiculation, and bronchial cutoff signs) can be obtained, and then, the sum (total points) of four “points” can be converted into the risk probability of lymph node metastasis. **(B)** The ROC curve of the nomogram. The area under the ROC curve (AUC) was used to assess the predictive performance of the model.

As shown in **Figure 3A**, in this study, we established a nomogram that could predict the probability of lymph node metastasis in PSILA preoperatively, based on solid part size, bronchial cutoff sign, and spiculation sign and CA199. Both the ROC curve and the C-index were used to assess the predictive power of the nomogram. As shown in **Figure 3B**, the nomogram has good predictive ability, with AUC of 0.858 and C-index of 0.857. In addition, we also found the best cutoff (0.027) for the model to predict the lymph node metastasis rate by the Youden index. That is, when the probability of lymph node metastasis obtained by nomogram exceeds 0.027, the patient is more likely to develop lymph node metastasis.

### Local validation of the IASLC novel grading system

**Figure 4:**
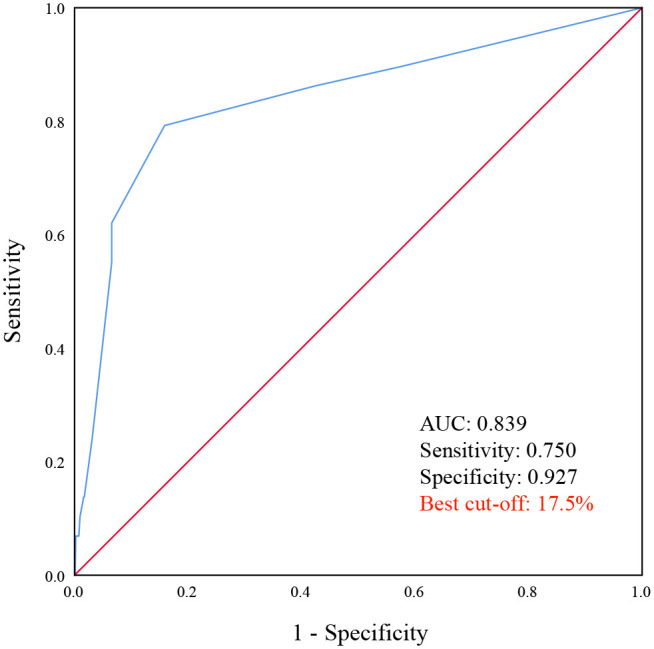
ROC curve of high-grade patterns for lymph node metastasis using local data. A cutoff of 17.5% for high-grade patterns was the value that offered the best combination of sensitivity and specificity of the curve (0.75 and 0.927, respectively), resulting in AUC of 0.839 for lymph node metastasis.

In addition to intra- and intertumoral heterogeneity, regional and ethnic differences in lung cancer cannot be ignored. The IASLC novel grading system for invasive lung adenocarcinoma was developed and validated based on European patients; however, its applicability to different Asian patients remains to be extensively studied. Therefore, in this study, we also explored the applicability of the new grading system using local data. In the new grading system, 20% was proposed as the optimal cutoff for the proportion of high-grade patterns with poor prognosis, and in this study (as shown in **Figure 4**), we determined that the optimal cutoff proportion of high-grade patterns by the Youden index was 17.5%. Based on the 5% increment recommended by WHO for the semi-quantitative assessment of pathological patterns of lung tumor (12), our results (17.5%) are nearly consistent with the new grading system (20%).

## Discussion

For non-early resectable NSCLC, radical and lymph node dissections are standard treatment. However, for early NSCLC, especially GGO and part-solid lung nodule, lobotomy or systematic lymph node dissection may be too aggressive due to the low lymph node metastasis rate. Obviously, this is a problem that needs to be solved: on the one hand, adequate lymph node dissection is beneficial to identify those potentially metastatic “pseudo-early” lung cancers (17), so as to accurately perform lymph node staging, and provide patients with adequate postoperative adjuvant therapy; on the other hand, for early-stage lung cancer without lymph node metastasis, it is meaningful to reduce trauma and maximize the preservation of lung structure and function. Therefore, in the era of precision medicine, research on preoperative lymph node staging as accurately as possible to guide intraoperative lymph node dissection decisions is warranted.

In recent years, the application of radiomics in identifying the invasiveness of subsolid lung cancers has been extensively studied. Studies have shown that, as an important component of TNM staging, tumor size (T staging) <6 mm tend to have a malignancy probability of <1%, while nodules between 6 and 8 mm have a malignancy probability of 1%–2% (18). Since most early-stage lung cancers often appear as lung adenocarcinomas with ground-glass components, increasing studies have shown that the solid part size is a better predictor of lung nodule invasiveness and patient prognosis than tumor size (19–22). Therefore, in the eighth edition of the TNM staging, it is recommended to define the T staging of part-solid lung cancer based on the solid part size (13). Moreover, some scholars found that CTR is also a reliable indicator for predicting lymph node metastasis in PSILA (23, 24). However, there were also studies based on a large number of cases that revealed that, although both tumor size and CTR can predict the aggressiveness of PSILA, they were not associated with lymph node metastasis and patients’ prognosis (2, 25, 26). In this study, we found that all of tumor size, solid part size, and CTR were highly associated with lymph node outcome of PSILA, but solid part size had significantly better predictive ability in predicting lymph node metastasis. Therefore, we chose solid part size for subsequent modeling. Other studies have shown that radiological features of pulmonary nodules, such as signs of vocule, spiculation, lobulation, pleural retraction, air bronchogram, bronchial cutoff, and vascular cluster, are often indicative of a high degree of malignancy and can therefore be used as predictors of poor prognosis in patients (27–31). In this study, we revealed that spiculation and bronchial cutoff sign on preoperative CT were independent risk factors for lymph node metastasis. Therefore, spiculation and bronchial cutoff sign were candidate indicators for modeling in this study.

In addition, a variety of serum tumor markers have been reported to have significant value in the diagnosis and treatment of multiple cancers. For instance, CEA always functioned as an adhesion molecule to promote metastasis of cancers (32). TPS has prognostic significance for survival in patients with advanced NSCLC, independent from performance status and stage of disease (33). CA199 is a tumor-associated oligosaccharide antigen on cell membrane, which has been reported as the most sensitive marker for pancreatic cancer and is also highly expressed in many other cancers (34–36). In this study, we explored the association between preoperative tumor markers in serum and lymph node metastasis in PSILA and found that patients with high CA199 had a significant lymph node metastasis rate compared to those with low CA199. Therefore, CA199 was also selected to construct a model to preoperatively predict lymph node metastasis.

Considering the controversy over the scope of intraoperative lymph node dissection in early lung cancer and the instability of a single indicator as a predictor, in this study, the clinical data of 960 cases of PSILA were reviewed. Univariate and multivariate logistic regressions were used to screen out clinicopathological factors, solid part size, CA199, spiculation, and bronchial cutoff sign, which were significantly correlated with lymph node metastasis of PSILA. Based on this, a nomogram was established for preoperative prediction of lymph node metastasis in PSILA. As a result, the nomogram had a good ability to predict lymph node metastasis (AUC, 0.858; C index, 0.857). In order to make the nomogram more convenient to use, we applied the Jorden index to find the optimal cutoff value of the positive probability of lymph node predicted by the model, which is 0.027. This means that, when the risk value obtained by the model exceeds 0.027, the patient is highly likely to suffer lymph node metastasis. Therefore, thorough lymph node dissection is necessary during the operation, even if the disease is in the early clinical stage.

In addition, we used local data of PSILA to confirm that the IASLC novel grading system is indeed superior to conventional predominant pattern-based groups in stratifying the prognosis of lung adenocarcinoma. Meanwhile, the optimal cutoff value (17.5%) of high-grade patterns screened out based on local data is basically close to that (20%) in the new grading system. This verifies the effectiveness and wide applicability of the new grading system for prognostic stratification of patients and also indicates the relative non-bias of the data in this study.

Studies have also shown that the maximum standard uptake value (SUVmax) of lung nodules in PET-CT can be used as an important preoperative factor to predict the pathological malignancy and prognosis of lung adenocarcinoma. Lung nodules with high SUVmax, even GGO-dominated nodules, are often malignant and often accompanied by lymph node metastasis (24, 37–39). A multicenter study of 502 patients with stage IA lung adenocarcinoma found that patients with SUVmax <1.5 could not undergo systematic lymph node dissection, even if the tumor reached 3 cm (40). Obviously, SUVmax is also very effective in predicting lymph node metastasis. However, it has to be mentioned that in developing countries and more remote areas, expensive PET-CT is out of reach for patients compared to cheap and easy thin-slice CT. In this study, since the solid part size, CA199, spiculation, and bronchial cutoff sign used for modeling are all clinically accessible indicators, we believe that this model has great clinical applicability, although the spiculation and bronchial cutoff sign require doctors to have certain lung image recognition skills. Of course, there are some limitations. Although the amount of data in this study is large, as a single-center retrospective study, it is naturally biased. Therefore, future validation of more patients and multicenter data is necessary.

## Conclusion

In this study, the reproducibility of the IASLC novel grading system for invasive lung adenocarcinoma was verified. In addition, we successfully built a nomogram based on diverse preoperative factors, providing a reference for intraoperative lymph node dissection, and again validated the IASLC grading system in terms of predicting the risk of LN metastasis.

## Data availability statement

The raw data supporting the conclusions of this article will be made available by the authors, without undue reservation.

## Ethics statement

This study was approved by an institutional review board (Tianjin Medical University Cancer Institute & Hospital review board bc2022082). Informed consents were waived because it was a retrospective study.

## Author contributions

ZG, XW, and ZZ: conceptualization. ZG, XW, and TZ: data curation and original draft writing. ZG, XW, TZ, and MZ: formal analysis. ZZ: funding acquisition. ZG, ZW, TZ, and MZ: methodology, draft review, and revision. ZG and ZZ: project administration and supervision. All authors contributed to the article and approved the submitted version.

## Conflict of interest

The authors declare that the research was conducted in the absence of any commercial or financial relationships that could be construed as a potential conflict of interest.

## Publisher’s note

All claims expressed in this article are solely those of the authors and do not necessarily represent those of their affiliated organizations, or those of the publisher, the editors and the reviewers. Any product that may be evaluated in this article, or claim that may be made by its manufacturer, is not guaranteed or endorsed by the publisher.

## Supplementary material

Supplementary Figure 1 Comparison of tumor size, solid part size, and CTR to predict lymph node metastasis in PSILA. The area under the ROC curve (AUC) was used to assess the predictive performance of different indicators.
