# Supplementary figures and images for: A predictive nomogram for lymph node metastasis in part-solid invasive lung adenocarcinoma: A complement to the IASLC novel grading system
Source: Front Oncol. 2022 Aug 15;12:916889. doi: 10.3389/fonc.2022.916889 (PMC9423719; doi:10.3389/fonc.2022.916889)

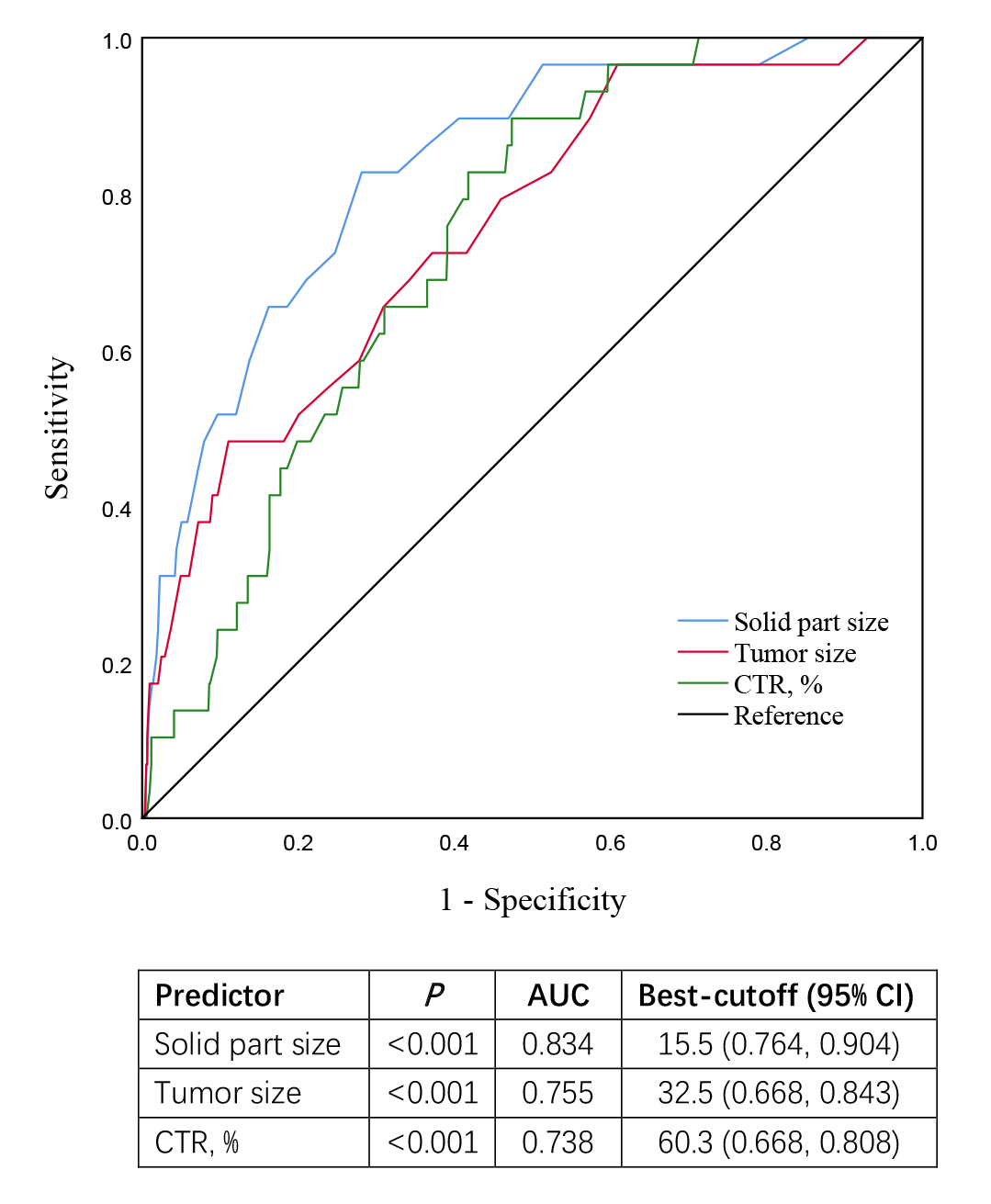

Supplement: Supplementary Figure 1 — Comparison of tumor size, solid part size, and CTR to predict lymph node metastasis in PSILA. The area under the ROC curve (AUC) was used to assess the predictive performance of different indicators. [file Image_1.tif]
